# A Hybrid Pathfinder Optimizer for Unconstrained and Constrained Optimization Problems

**DOI:** 10.1155/2020/5787642

**Published:** 2020-05-29

**Authors:** Xiangbo Qi, Zhonghu Yuan, Yan Song

**Affiliations:** ^1^School of Mechanical Engineering, Shenyang University, Shenyang 110044, China; ^2^School of Physics, Liaoning University, Shenyang 110036, China

## Abstract

Hybridization of metaheuristic algorithms with local search has been investigated in many studies. This paper proposes a hybrid pathfinder algorithm (HPFA), which incorporates the mutation operator in differential evolution (DE) into the pathfinder algorithm (PFA). The proposed algorithm combines the searching ability of both PFA and DE. With a test on a set of twenty-four unconstrained benchmark functions including both unimodal continuous functions, multimodal continuous functions, and composition functions, HPFA is proved to have significant improvement over the pathfinder algorithm and the other comparison algorithms. Then HPFA is used for data clustering, constrained problems, and engineering design problems. The experimental results show that the proposed HPFA got better results than the other comparison algorithms and is a competitive approach for solving partitioning clustering, constrained problems, and engineering design problems.

## 1. Introduction

The main characteristics of metaheuristic algorithm are that there are few parameters and operators. It is easy to apply them to actual problems. Every metaheuristic algorithm has its advantages and disadvantages. For instance, artificial bee colony (ABC) algorithm is a relatively new metaheuristic algorithm inspired by the foraging behaviors of honey bee colony [1]. Because ABC is easy to implement, has few control parameters, and possesses better optimizing performance [2], ABC has been successfully applied to solve optimization problems [3, 4]. However, with the increase of the dimensionality of the search space, ABC has a poor convergence behavior. The reason for that is because the ABC algorithm relies on the exchange of information between individuals. But each individual exchanges information on only one dimension with a random neighbor in each searching process. Yang carries out a critical analysis of the ABC algorithm by analyzing the way to mimic evolutionary operators [5]. In essence, operators in ABC belong to the mutation operator. So ABC shows a slower convergence speed. Like ABC algorithm, artificial butterfly optimization (ABO) algorithm is also an algorithm to simulate biological phenomena. The ABO algorithm simulates mate-finding strategy of some butterfly species and is tested on various benchmarks [6]. However, “No free lunch” theorems [7] suggest that one algorithm could not possibly show the best performance for all problems. Many strategies including improving existing algorithms or studying new algorithms can get better optimization effects. These strategies include opposition-based learning, chaotic theory, topological structure-based method, and hybridizing strategy. The strategy of hybridizing heterogeneous biological-inspired algorithms is a good way to balance the exploration and exploitation [8]. In order to add the diversity of the bat swarm, a hybrid HS/BA method adding pitch adjustment operation in HS to the BA method is proposed [9]. The hybridization of nature-inspired algorithms evolved as a solution necessary in overcoming certain shortcomings observed during the use of classical algorithms [10]. Good convergence requires clever exploitation at the right time and at the right place, which is still an open problem [11].

Pathfinder algorithm (PFA) is a relatively new metaheuristic algorithm inspired by the collective movement of animal group and mimics the leadership hierarchy of swarms to find best food area or prey [12]. PFA provides superior performance in some optimization problems. However, when the dimension of a problem is extremely increased, the performance of this method decreases because PFA mainly relies on mathematical formulas. The strategy of hybridizing heterogeneous biological-inspired algorithms can avoid the shortcomings of single algorithm because of increasing the individual information exchange. The differential evolution (DE) algorithm which is proposed by Storn and Price [13] performs very well in convergence [14]. In particular, DE has a good performance on searching the local optima and good robustness [15]. In view of the fast convergence speed of DE, the mutation operator in DE is incorporated into the PFA. Then, a hybrid pathfinder algorithm is proposed in this paper.

The rest of the paper is organized as follows.  Section 2 will introduce the canonical PFA.  Section 3 will present HPFA in detail.  Section 4 will give the details of the experiment for unconstrained problems. The experiment results are also presented and discussed in this section.  Section 5 introduces the data clustering problem and how the HPFA is used for clustering.  Section 6 will give the details of the experiment for constrained problems.  Section 7 will give the details of the experiment for engineering design problems.  Section 8 gives the conclusions.

## 2. The Canonical Pathfinder Algorithm

The canonical PFA mimics the leadership hierarchy of animal group to find best prey. In the PFA, the individual with the best fitness is called pathfinder. The rest members of the swarm which are called followers in this paper follow the pathfinder. The PFA includes three phases: initialization phase, pathfinder's phase, and followers' phase.

In the initialization phase, the algorithm randomly produces a number of positions according to equation (1) in the search range. After that, the fitness values of the positions are calculated. The individual with the best fitness is selected as the pathfinder:

(1) $$\begin{array}{c} x_{i,j}=x_{j}^{\min}+\text{rand}\left[0,1\right]\left(x_{j}^{\max}-x_{j}^{\min}\right), \end{array}$$

where *i*∈ [1, 2,…, *SN*] and *j* ∈   [1, 2,…, *D*]. *SN* is the number of swarm. *D* is the number of parameters of the optimization problem. *x*_*j*_^min^ is the lower boundary value of the *j*th parameter and *x*_*j*_^max^ is the upper boundary value of the *j*th parameter.

In the pathfinder's phase, the position of the pathfinder is updated using equation (2). A greedy selection strategy is employed by comparing the fitness value of the new position of the pathfinder and the old one:

(2) $$\begin{array}{c} x_{p}^{k+1}=x_{p}^{k}+2r_{3}\left(x_{p}^{k}-x_{p}^{k-1}\right)+A, \end{array}$$

where *x*_*p*_ is the position vector of the pathfinder, *k* is the current iteration, and *r*_3_ is a random vector uniformly generated in the range of [0, 1]. *A* is generated using the following equation:

(3) $$\begin{array}{c} A=u_{2}e^{-2k/E}, \end{array}$$

where *u*_2_ is a random vector range in [−1, 1], *k* is the current iteration, and *E* is the maximum number of iterations.

In the followers' phase, the position of each follower is updated using equation (4). A greedy selection strategy is employed by comparing the fitness value of the new position of the follower and the old one. If the fitness of the follower with the best fitness is better than that of the pathfinder, the pathfinder is replaced with the follower:

(4) $$\begin{array}{c} x_{i}^{k+1}=x_{i}^{k}+R_{1}\left(x_{j}^{k}-x_{i}^{k}\right)+R_{2}\left(x_{p}^{k}-x_{i}^{k}\right)+\varepsilon , \end{array}$$

(5) $$\begin{array}{c} R_{1}=\alpha r_{1}, \end{array}$$

(6) $$\begin{array}{c} \varepsilon =\left(1-\frac{k}{E}\right)u_{1}D_{ij}, \end{array}$$

(7) $$\begin{array}{c} D_{ij}=x_{i}-x_{j}, \end{array}$$

where *x*_*i*_ is the position vector of the *i*th follower, *x*_*j*_ is the position vector of the *j*th follower, *k* is the current iteration, and *E* is the maximum number of iterations. *r*_1_ and *r*_2_ are random values generated in the range of [0,1]. *α* and *β* are random values generated in the range of [1, 2], and *D*_*ij*_ is the distance between the *i*th follower and the *j*th follower.

The termination condition of the PFA may be the maximum cycles or the maximum function evaluation.

## 3. The Hybrid Pathfinder Algorithm

The commonly used hybrid methods mainly include series and parallel. The series method refers to the optimization operation for all members of swarm in the evolution of each generation. The series method is used in the proposed hybrid algorithm. In DE, the differential mutation operator is the main operation. In view of the fast convergence speed of DE, the mutation operator in DE is incorporated into the PFA to form a new hybrid pathfinder algorithm (HPFA). In HPFA, the rest parts are the same as the canonical PFA except a mutation phase is added after the followers' phase. The pseudocode of HPFA is listed in Algorithm 1. The steps of the mutation phase are given below. For each follower *x*_*i*_ in the swarm, do the following steps:

  Step 1: select three different followers *x*_*r*_, *x*_*p*_, and *x*_*q*_ from the followers. The three values *r*, *p*, and *q* are not equal to *i.*
  Step 2: for each dimension in *D*, produce a new position vector depending on CR. CR is a probability in the range of [0, 1]. The new position vector is produced according to equation (8). The new position vector *v*_*ij*_ is determined by changing one dimension of *x*_*i*_ and is set to its boundary value if exceeding its predetermined boundaries:

(8) $$\begin{array}{c} v_{ij}=x_{rj}+F\left(x_{pj}-x_{qj}\right), \end{array}$$

  where *i*, *r*, *p*, *q* are four different integers generated by random permutation, *F* is the differential weight in the range of [0, 2]. *j* is a randomly selected dimension index between [1, *D*].
  Step 3: calculate the fitness of the new position vector.
  Step 4: a greedy selection strategy is employed by comparing the fitness value of the new position vector and the original one. If the fitness of the new position vector is better than the original one, it will replace the original one. Otherwise, the original one does not make any change.

## 4. Unconstrained Benchmark Problems

For the ease of visualization, we have implemented all algorithms using Matlab for various test functions. In order to compare the different algorithms fairly, we use a number of function evaluations (FEs) as a measure criterion in this paper.

### 4.1. Benchmark Functions

Evolutionary algorithms are usually experimentally assessed through various test problems because an analytical assessment of their behavior is very complex [16]. The twenty-four benchmark functions are widely adopted by other researchers to test their algorithms in many works [2, 17, 18]. In this paper, all functions used their standard ranges. These benchmark functions totaling twelve diverse and difficult minimization problems comprise unimodal continuous functions (*f*_1_ − *f*_8_), multimodal continuous functions (*f*_9_ − *f*_16_), and composition functions (*f*_17_ − *f*_24_). The formulas of these functions are presented in Table 1. Functions *f*_13_ − *f*_16_ are four rotated functions employed in Liang's work [19]. In the rotated functions, a rotated variable *y*, which is produced by the original variable *x* left multiplied an orthogonal matrix, is used to calculate the fitness (instead of *x*). The orthogonal matrix is generated according to Salomon's method [20]. Functions *f*_17_ − *f*_24_ are eight composition functions.

These composition functions were specifically designed for the competition and comprise the sum of three of five unimodal and/or multimodal functions, leading to very challenging properties: multimodal, nonseparable, asymmetrical, and with different properties around different local optima.

### 4.2. Parameter Study

The choice of parameters can have an effect on the performance of an algorithm. QPSO has demonstrated the high potential for setting parameters of optimization methods [21]. Computational intelligence methods have demonstrated their ability to monitor complex large scale systems, but the selection of optimal parameters for efficient operation is very challenging [22]. The parameter CR and parameter *F* are two parameters in HPFA. In order to analyze the impact of the two parameters, we do the following experiments. In all experiments, the population size of all algorithms was 100. The maximum evaluation count on dimensions 20 is 100,000.

Four continuous benchmark functions, Sphere 20D, Zakharov 20D, Sumsquares 20D, and Quadric 20D are employed to investigate the impact of parameter CR and parameter *F*. Set CRratio_e_ and *F* equal to different values and all the functions run 20 sample times. It is worth noting that the interval of CRratio_e_ and *F* is continuous and has numerous values. Here, three different values of the two parameters are used. The experimental results in terms of mean values and standard deviation of the optimal solutions over 30 runs are listed in Tables 2–5. From the results, we can find that HPFA with CRratio_e_ equal to 0.9 and *F* equal to 0.1 performs best on all four functions. According to the results, we chose CR equal to 0.9 and *F* equal to 0.1 for the next experiments.

### 4.3. Comparison with Other Algorithms

In order to compare the performance of HPFA, PFA [12], differential evolution (DE) [13], canonical PSO with constriction factor (PSO) [23], and cooperative PSO (CPSO) [24] were employed for comparison. PSO is a classical population-based paradigm simulating the foraging behavior of social animals. CPSO is a cooperative PSO model, cooperatively coevolving multiple PSO subpopulations. In addition, a set of twelve well-known benchmark functions were used in this experiment.

#### 4.3.1. Experiment Sets

The population size of all algorithms was 100. The maximum evaluation count on dimensions 30 is 100,000. In order to do meaningful statistical analysis, each algorithm runs for 30 times and takes the mean value and the standard deviation value as the final result. For the specific parameters for comparison algorithms, we follow parameter settings of the original literature studies. For CPSO and PSO, the learning rates *C*1 and *C*2 were both set as 2.05. The constriction factor *X* = 0.729. The split factor for CPSO is equal to the dimensions. In DE, single-point crossover is employed, the crossover rate is 0.95, and *F* is 0.1.

All algorithms were implemented in Matlab R2010a using a computer with Intel Core i5-2450M CPU, 2.5 GHz, 2 GB RAM. The operating system of the computer is Windows7.

#### 4.3.2. Experimental Results and Analysis

**Figure 1:**
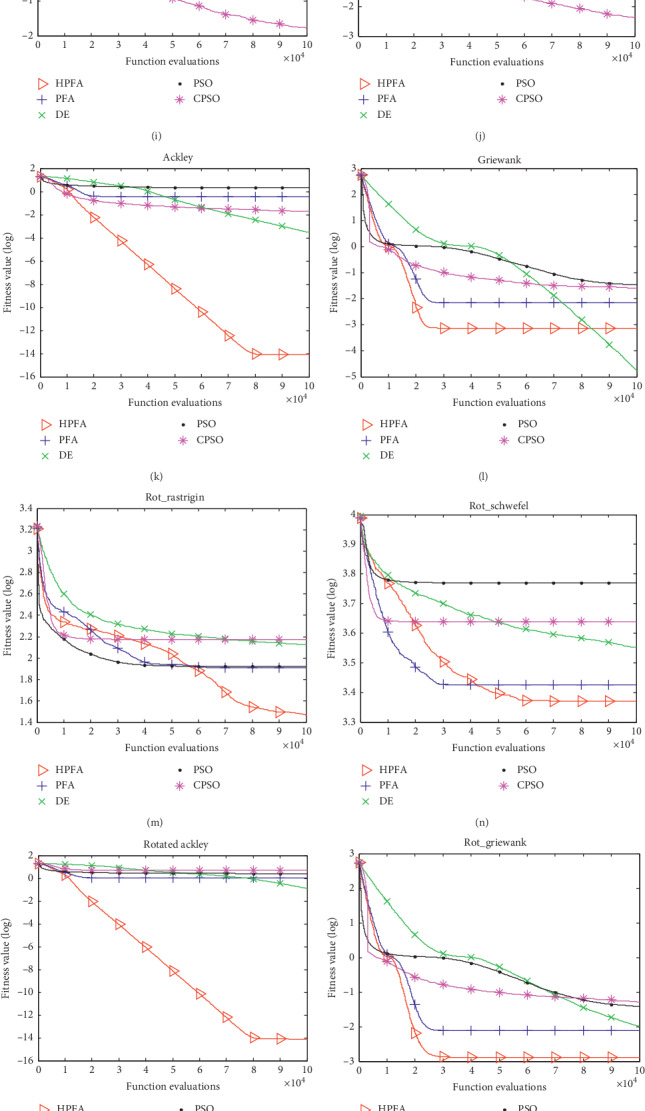
The mean best function value profiles of HPFA, PFA, DE, PSO, and CPSO. (a) (*f*_1_), (b) (*f*_2_), (c) (*f*_3_), (d) (*f*_4_), (e) (*f*_5_), (f) (*f*_6_), (g) (*f*_7_), (h) (*f*_8_), (i) (*f*_9_), (j) (*f*_10_), (k) (*f*_11_), (l) (*f*_12_), (m) (*f*_13_), (n) (*f*_14_), (o) (*f*_15_), (p) (*f*_16_), (q) (*f*_17_), (r) (*f*_18_), (s) (*f*_19_), (t) (*f*_20_), (u) (*f*_21_), (v) (*f*_22_), (w) (*f*_23_), and (x) (*f*_24_).

The experimental results, including the mean and the standard deviation of the function values obtained by the five algorithms with 30 dimensions, are listed in Table 6. The best values obtained on each function are marked as bold. Rank represents the performance order of the five algorithms on each benchmark function. It is obvious that HPFA performed best on most functions. The mean best function value profiles of the five algorithms with 30 dimensions are shown in Figure 1.

*(1) Continuous Unimodal Functions*. On Sphere function, the performance order for the five intelligent algorithms is HPFA > PFA > DE > CPSO > PSO. The result achieved by HPFA was improved continually and got the best value, seen from Figure 1(a). The performance of DE, PSO, and CPSO deteriorates in optimizing this function. HPFA has very strong solving performance on Sphere function. HPFA and these three algorithms differ by about 30 orders of magnitude of solution quality.

On Sinproblem function, the performance order for the six intelligent algorithms is HPFA > DE > CPSO > PFA > PSO. The performance of HPFA is much similar to it on Sphere. The result achieved by HPFA was improved continually. PFA and PSO converged very fast at the beginning and then trapped in local minimum. CPSO and DE converged continually, but the speed of convergence was slow, seen from Figure 1(d). The performance of PFA, PSO, CPSO, and DE deteriorates in optimizing this function.

On Sumsquares function, the performance order for the five intelligent algorithms is HPFA > PFA > DE > CPSO > PSO. The performance of HPFA is much similar to it on Sphere and Sinproblem. The result achieved by HPFA was improved continually. DE converged slowly, seen from Figure 1(e). The performance of PSO and CPSO deteriorates in optimizing this function.

On Schwefel2.22 function, the performance order for the five intelligent algorithms is HPFA > PFA > DE > CPSO > PSO. The performance of PSO and CPSO deteriorates in optimizing this function. The result achieved by PFA and HPFA was improved continually. Finally, HPFA got better results than PFA, seen from Figure 1(h).

*(2) Continuous Multimodal Functions*. On Ackley function, the performance order for the five intelligent algorithms is HPFA > DE > CPSO > PFA > PSO. The Ackley function poses a risk for optimization algorithms, so many algorithms are trapped in one of its many local minima. The Ackley function is widely used for testing optimization algorithms. The performance of DE, PSO, CPSO, and PFA deteriorates in optimizing this function. HPFA has a much stronger global searching ability, seen from Figure 1(k). The solution of HPFA is about 11 orders higher than that of DE.

The multimodal functions *f*_13_–*f*_16_ are regarded as the most difficult functions to optimize since the number of local minima increases exponentially as the function dimension increases.

On Rot_rastrigin function, the performance order for the five intelligent algorithms is HPFA > PFA > PSO > DE > CPSO. The result achieved by HPFA was improved continually and got the best result, seen from Figure 1(m). PFA and PSO converged to a local minimum value at about 40,000 FEs. CPSO and DE perform worse than PSO and PFA.

On Rot_schwefel function, the performance order for the five intelligent algorithms is HPFA > PFA > DE > CPSO > PSO. CPSO and PSO converged fast at first, but it becomes trapped in a local minimum very soon. DE converged continually, but the speed of convergence was slow. Finally, HPFA got better results than PFA, seen from Figure 1(n).

On Rot_ackley function, the performance order for the five intelligent algorithms is HPFA > DE > PFA > PSO > CPSO. The solution of HPFA is about 14 orders higher than that of DE. At the very beginning, PFA, CPSO, DE, and PSO converged very fast and then trapped in local minimum. The result achieved by HPFA was improved continually and got the best result, seen from Figure 1(o).

On Rot_griewank function, the performance order for the five intelligent algorithms is HPFA > PFA > DE > PSO > CPSO. CPSO, PSO, and DE converged very slowly. HPFA and PFA converged continually and then trapped in local minimum, but HPFA performed better than PFA, seen from Figure 1(p).

*(3) Composition Function*. On Composition Function 4, Composition Function 5, Composition Function 7, and Composition Function 8, HPFA got the best result.

From the above analysis, we can observe that the ability of exploiting the optimum of HPFA is very strong. HPFA seemed to have the ability of continual improving especially on Sphere, Sinproblem, Sumsquares, Schwefel2.22, Ackley, Rotated Rastrigin, Rotated Schwefel, Rotated Ackley, and Rotated Griewank.

### 4.4. Statistical Analysis

It is obvious that HPFA got the best ranking with a dimension of 30. Statistical evaluation of experimental results has been considered an essential part of validation of new intelligent methods. The Iman–Davenport and Holm tests are nonparametric statistical methods and used to analyze the behaviors of evolutionary algorithms in many recent works. The Iman–Davenport and Holm tests are used in this section. Details of the two statistical methods are introduced in reference [25]. The results of the Iman–Davenport test are shown in Table 6. The values are distributed according to *F*-distribution with 4 and 92 degrees of freedom. The critical values are looked up in the *F*-distribution table with a level of 0.05. As can be seen in Table 7, the Iman–Davenport values are larger than their critical values, which means that significant differences exist among the rankings of the algorithms.

Holm test was employed as a post hoc procedure. HPFA was chosen as the control algorithm. The results of Holm tests are given in Table 8. The *α*/*i* values listed in the tables are with a level of 0.05.

HPFA got the best ranking and is the control algorithm. As seen in Table 8, the *p* values of PSO, CPSO, DE, and PFA are smaller than their *α*/*i* values, which means that equality hypotheses are rejected and significant differences exist between these five algorithms and the control algorithm.

### 4.5. Algorithm Complexity Analysis

**Figure 2:**
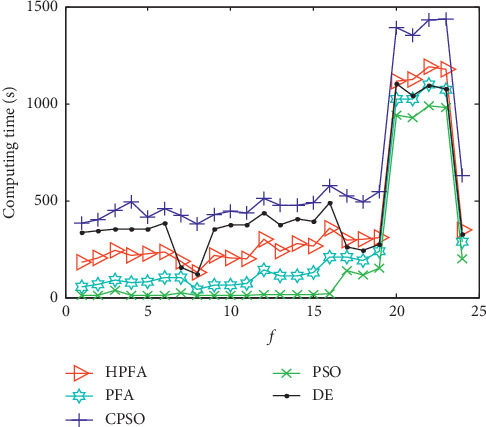
Computing time of all algorithms on different problems.

In many heuristic algorithms, most of the computation is spent on fitness evaluation in each generation. The computation cost of one individual is associated with the test function complexity. It is very difficult to give a brief analysis in terms of time for all algorithms. Through directly evaluating the algorithmic time response on different benchmark functions (*f*_1_−*f*_24_), the total computing time for 30 sample runs of all algorithms is given in Figure 2. From the results, it is observed that CPSO takes the most computing time in all compared algorithms. PSO takes the least computing time in all compared algorithms. In summary, it is concluded that, compared with other algorithms, HPFA requires less computing time to achieve better results.

## 5. Application to Data Clustering

Data clustering is a kind of typical unsupervised learning, which is used to divide the samples of unknown categories. Clustering algorithm is widely used in banking, retail, insurance, medical, military, and other fields. Many clustering algorithms including hierarchical methods, partitioning methods, and density-based methods are proposed. In this paper, we mainly focus on partitioning clustering. Given a set of *n* data objects and the number of clusters *w* to be formed, the partitioning method divides the set of objects into *w* parts. Each partition represents a cluster. The final clustering will optimize a partition criterion, so that the objects in a cluster are similar, while the objects in different clusters are not. Generally, the total mean square quantization error (MSE) is used as the standard measure function for partitioning. Let *X*=(*x*_1_, *x*_2_,…, *x*_*n*_) be a set of *n* data and *C*=(*c*, *c*,…, *c*_*w*_) be a set of *w* clusters. The following equation gives the definition of MSE. Minimizing this objective function is known to be an NP-hard problem (even for *K* = 2) [26]:

(9) $$\begin{array}{c} \text{Perf}\left(X,C\right)=\sum_{i=1}^{n}\min\left\{\left.x_{i}-c_{j}^{2}\right|j=1,2,\ldots ,w\right\}, \end{array}$$

(10) $$\begin{array}{c} x_{i}-c_{j}=\sqrt{\sum_{m=1}^{P}{\left(x_{i,m}-c_{j,m}\right)}^{2}}, \end{array}$$

where *w* is the number of clusters *i* ∈ [1, *n*]. *c*_*j*_ denotes a clustering center. *n* denotes the size of the dataset. Each data object *x*_*i*_ in the dataset has *p* features. *x*_*i*_ − *c*_*j*_ denotes the Euclidean distance between *x*_*i*_ and *c*_*j*_.

### 5.1. The HPFA Algorithm for Data Clustering

In HPFA for clustering, each individual denotes a set of cluster centers according to equation (11). According to equation (12), each artificial butterfly can be decoded to a cluster center:

(11) $$\begin{array}{c} X_{i}=\left\{x_{1},x_{2},\ldots ,x_{p},x_{p+1},\ldots ,x_{w\times p}\right\}, \end{array}$$

where *w* is the number of clusters and *p* is the number of features of the data clustering problem:

(12) $$\begin{array}{c} C_{m}=\left\{x_{\left(m-1\right)\times p+1},x_{\left(m-1\right)\times p+2},\ldots ,m_{m\times p}\right\},\;m=1,2,\ldots ,w. \end{array}$$

According to equation (9), the fitness of each individual can be calculated.  Algorithm 2 gives the main steps of the fitness function.

### 5.2. Experiment Sets

To verify the performance of the HPFA algorithm for data clustering, PFA, CPSO, and PSO are used to compare on several datasets, including Glass, Wine, Iris, and LD. These datasets are selected from the UCI machine learning repository [27].

In order to provide meaningful statistical analyses, each algorithm is run 30 times independently. The experimental results include the mean value and the standard deviation value. The population size of the four algorithms is set to 20. The maximum number of evaluations is 10000. Parameters for HPFA, PFA, CPSO, and PSO are the same with ones in Section 4.

### 5.3. Results and Analysis

**Figure 3:**
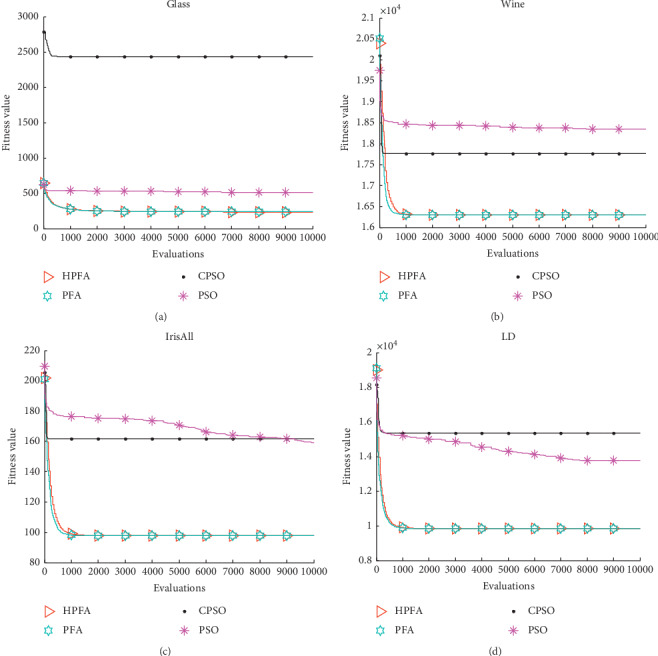
The mean minimum total within-cluster variance profiles of HPFA, PFA, CPSO, and PSO. (a) Glass data, (b) Wine data, (c) Iris data, and (d) LD data.

Table 9 gives the results obtained by HPFA, PFA, CPSO, and PSO.  Figure 3 shows the mean minimum total within-cluster variance profiles of HPFA, PFA, CPSO, and PSO.

The Glass dataset consists of 214 instances characterized by nine attributes. There are two categories in the data. As seen from Figure 3(a), CPSO converged quickly from the beginning and trapped a local minimum. HPFA and PFA converged continually.

The Wine dataset consists of 178 objects characterized by thirteen features. There are three categories in the data. As seen from Figure 3(b), CPSO and PSO converged quickly from the beginning and trapped a local minimum. PFA and HPFA converged continually before about 1000 FEs.

The Iris dataset consists of 150 objects characterized by four features. There are three categories in the data. As seen from Figure 3(c), CPSO converged more quickly and trapped a local minimum obviously. PSO converged slowly.

The LD dataset consists of 345 objects characterized by six features. There are two categories. With the LD dataset, CPSO trapped a local minimum obviously at the very beginning. PSO converged slowly, but PSO got better results than CPSO, as seen from Figure 3(d).

The performance of HPFA and PFA is much similar to Wine, Iris, and LD, but HPFA got the best result. Experimental results given in Table 9 show that HPFA outperforms the other clustering algorithms in terms of the quality of the solutions for four datasets including Glass, Wine, Iris, and LD.

## 6. Constrained Benchmark Problems

Experimental sets are as follows: the population size was 100 for HPFA. In order to do meaningful statistical analyses, each algorithm runs 25 times and takes the mean value and the standard deviation value as the final result. “FEs,” “SD,” and “NA” stand for number of function evaluations, standard deviation, and not available, respectively. The mathematical formulations for constrained benchmark functions (problems 1–4) are given in Appendixes A–D.

### 6.1. Constrained Problem 1

In order to compare the performance of HPFA on constrained problem 1 (see Appendix A), WCA [28], IGA [29], PSO [30], CPSO-GD [31], and CDE [32] were employed for comparison.  Table 10 gives the best results obtained by HPFA, WCA, and IGA.  Table 11 gives the comparison of statistical results obtained from various algorithms for constrained problem 1. As shown in Table 11, in terms of the number of function evaluations, HPFA shows superiority to other algorithms.

### 6.2. Constrained Problem 2

In order to compare the performance of HPFA on constrained problem 2 (see Appendix B), WCA [28], PSO [30], PSO-DE [30], GA1 [33], HPSO [34], and DE [35] were employed for comparison.  Table 12 gives the best results obtained by GA1, WCA, and HPFA.  Table 13 gives the comparison of statistical results obtained from various algorithms for constrained problem 2. As shown in Table 13, HPFA offered the best solution quality in less number of function evaluations for this problem. The proposed HPFA reached the best solution (−30665.5386) in 15,000 function evaluations.

### 6.3. Constrained Problem 3

In order to compare the performance of HPFA on constrained problem 3 (see Appendix C), WCA [28], PSO [30], PSO-DE [30], DE [35], and CULDE [36] were employed for comparison.  Table 14 gives the best results obtained by GA1, WCA, and HPFA.  Table 15 gives the comparison of statistical results obtained from various algorithms for constrained problem 3. As shown in Table 15, HPFA reached the best solution (−0.999989) in 100,000 function evaluations.

### 6.4. Constrained Problem 4

In order to compare the performance of HPFA on constrained problem 4 (see Appendix D), WCA [28], HPSO [34], PESO [37], and TLBO [38] were employed for comparison.  Table 16 gives the best results obtained by WCA and HPFA.  Table 17 gives the comparison of statistical results obtained from various algorithms for constrained problem 4. As shown in Table 17, HPFA reached the best solution (−1) in 5,000 function evaluations which is considerably less than other compared algorithms.

## 7. Engineering Design Problems

### 7.1. Three-Bar Truss Design Problem

In order to compare the performance of HPFA on the three-bar truss design problem (see Appendix E), WCA [28] and PSO-DE [30] were employed for comparison.  Table 18 gives the best results obtained by PSO-DE, WCA, and HPFA. The comparison of obtained statistical results for HPFA with previous studies including WCA and PSO-DE is presented in Table 19. As shown in Table 19, HPFA obtained the best mean value in 10,000 function evaluations which is superior to PSO-DE.

### 7.2. Speed Reducer Design Problem

In order to compare the performance of HPFA on speed reducer design problem (see Appendix F), WCA [28], PSO-DE [30], and HEAA [39] were employed for comparison.  Table 20 gives the best results obtained by HPFA, PSO-DE, WCA, and HEAA. The comparison of obtained statistical results for HPFA with previous studies including WCA, PSO-DE, and HEAA is presented in Table 21. As shown in Table 21, HPFA obtained the best mean value in 11,000 function evaluations which is superior to other considered algorithms.

### 7.3. Pressure Vessel Design Problem

In order to compare the performance of HPFA on pressure vessel design problem (see Appendix G), WCA [28], PSO [31], CPSO [31],and GA3 [40] were employed for comparison.  Table 22 gives the best results obtained by WCA, HPFA, CPSO, and GA3. The comparison of obtained statistical results for HPFA with previous studies including WCA, CPSO, and PSO is presented in Table 23. As shown in Table 23, HPFA obtained better mean value than PSO in 25,000 function evaluations.

### 7.4. Tension/Compression Spring Design Problem

In order to compare the performance of HPFA on tension/compression spring design problem (see Appendix H), WCA [28], CPSO [31], and GA3 [40] were employed for comparison.  Table 24 gives the best results obtained by WCA, HPFA, CPSO, and GA3. The comparison of obtained statistical results for HPFA with previous studies including WCA, CPSO, and GA3 is presented in Table 25. As shown in Table 25, HPFA obtained the best mean value in 22,000 function evaluations which is superior to WCA, CPSO, and GA3.

### 7.5. Welded Beam Design Problem

In order to compare the performance of HPFA on welded beam design problem (see Appendix I), WCA [28], CPSO [31], and GA3 [40] were employed for comparison.  Table 26 gives the best results obtained by WCA, HPFA, CPSO, and GA3. The comparison of obtained statistical results for HPFA with previous studies including WCA, CPSO, and GA3 is presented in Table 27. As shown in Table 27, HPFA obtained the best mean value in 22,000 function evaluations which is superior to WCA, CPSO, and GA3.

## 8. Conclusion

The strategy of hybridizing heterogeneous biological-inspired algorithms can avoid the shortcomings of single algorithm because of increasing the individual information exchange. This paper proposed a hybrid pathfinder algorithm (HPFA), in which the mutation operator in DE is introduced. To validate the performance of HPFA, abundant experiments on twenty-four unconstrained benchmark functions compared with PFA, CPSO, PSO, and DE are carried out. The numerical experimental results show that HPFA has a good optimizing ability on most benchmark functions and outperforms the original PFA and the other comparison algorithms. Then HFPA is used for data clustering. Real datasets selected from the UCI machine learning repository are used. The experimental results show that the proposed HPFA got better results than the other comparison algorithms on the four datasets.

Then HPFA is employed to solve four constrained benchmark problems and five engineering design problems. The experiment results show that HPFA obtained better solutions than the other comparison algorithms with less function evaluations on most problems. It proves that HPFA is an effective method for solving constrained problems. However, HPFA will still trap in local minimum on a few functions, which can be seen from the benchmark functions. Finding the features of functions which HPFA works not well on and improving the algorithm in solving these functions are the future work.

## Figures and Tables

**Algorithm 1:**
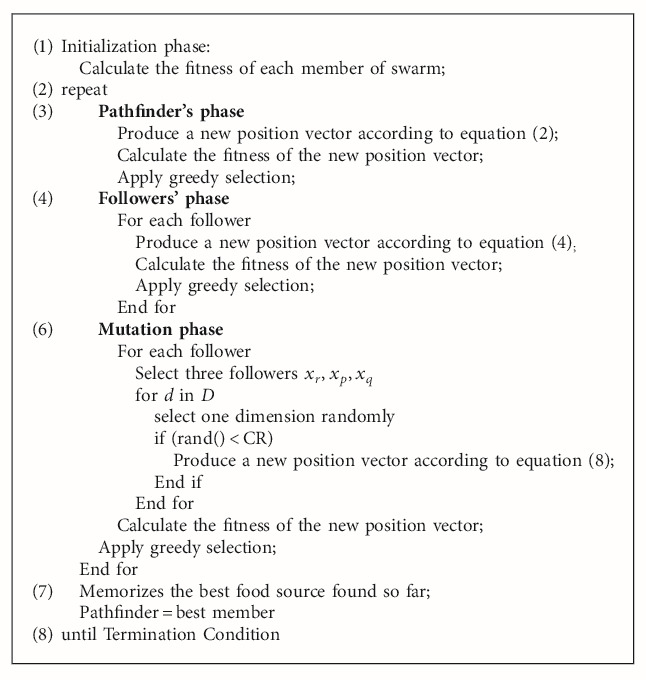
Pseudocode of HPFA.

**Algorithm 2:**
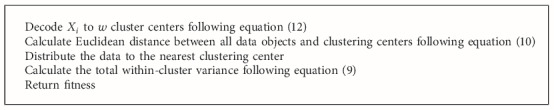
Pseudocode of fitness calculation.

**Table 1:** Benchmark functions.

Name	Function	Limits
Sphere (*f*_1_)	*f*_1_=∑_*i*=1_^*D*^*x*_*i*_^2^	[−5.12, 5.12]^*D*^
Rosenbrock (*f*_2_)	*f*_2_=∑_*i*=1_^*D*^(100(*x*_*i*_^2^ − *x*_*i*+1_)^2^+(1 − *x*_*i*_)^2^)	[−15,15]^*D*^
Quadric (*f*_3_)	*f*_3_=∑_*i*=1_^*D*^(∑_*j*=1_^*i*^*x*_*j*_)^2^	[−10,10]^*D*^
Sinproblem (*f*_4_)	*f*_*m*_=10 sin^2^ *πx*_1_+∑_*i*=1_^*D*−1^(*x*_*i*_ − 1)^2^(1+10 sin^2^ *πx*_*i*+1_)	[−10,10]^*D*^
	*f*_4_=*π*/*D*{*f*_*m*_+(*x*_*D*_ − 1)^2^}	
Sumsquares (*f*_5_)	*f*_5_=∑_*i*=1_^*D*^*ix*_*i*_^2^	[−10,10]^*D*^
Zakharov (*f*_6_)	*f*_6_=∑_*i*=1_^*D*^*x*_*i*_^2^+(∑_*i*=1_^*D*^0.5*ix*_*i*_)^2^+(∑_*i*=1_^*D*^0.5*ix*_*i*_)^4^	[−5,10]^*D*^
Powers (*f*_7_)	*f*_7_=∑_*i*=1_^*D*^|*x*_*i*_|^*i*+1^	[−1,1]^*D*^
Schwefel2.22 (*f*_8_)	*f*_8_=∑_*i*=1_^*D*^|*x*_*i*_|+∏_*i*=1_^*D*^|*x*_*i*_|	[−10,10]^*D*^
Rastrigin (*f*_9_)	*f*_9_=∑_*i*=1_^*D*^(*x*_*i*_^2^ − 10 cos(2*πx*_*i*_)+10)	[−15,15]^*D*^
Schwefel (*f*_10_)	$f_{10}=D\cdot 418.9829+\sum_{i=1}^{D}-x_{i}\sin\left(\sqrt{\left|x_{i}\right|}\right)$	[−500,500]^*D*^
Ackley (*f*_11_)	$f_{m}=20+e-20\;\exp\left(-0.2\sqrt{\left(1/D\right)\sum_{i=1}^{D}x_{i}^{2}}\right)$	[−32.768, 32.768]^*D*^
	*f*_11_=*f*_*m*_ − exp((1/*D*)∑_*i*=1_^*D*^cos(2*πx*_*i*_))	
Griewank (*f*_12_)	$f_{12}=1/4000\left(\sum_{i=1}^{D}x_{i}^{2}\right)-\left(\prod_{i=1}^{D}\cos\left(x_{i}/\sqrt{i}\right)\right)+1$	[−600,600]^*D*^
Rot_rastrigin (*f*_13_)	*f*_13_=*f*_5_(*y*), *y*=*M* × *x*	[−15,15]^*D*^
Rot_schwefel (*f*_14_)	*f*_14_=*f*_6_(*y*), *y*=*M* × *x*	[−500,500]^*D*^
Rot_ackley (*f*_15_)	*f*_15_=*f*_7_(*y*), *y*=*M* × *x*	[−32.768, 32.768]^*D*^
Rot_griewank (*f*_16_)	*f*_16_=*f*_8_(*y*), *y*=*M* × *x*	[−600,600]^*D*^
Composition Function 1 (*n* = 5, rotated) (*f*_17_)		[−100,100]^*D*^
Composition Function 2 (*n* = 3, unrotated) (*f*_18_)		[−100,100]^*D*^
Composition Function 3 (*n* = 3, rotated) (*f*_19_)		[−100,100]^*D*^
Composition Function 4 (*n* = 3, rotated) (*f*_20_)		[−100,100]^*D*^
Composition Function 5 (*n* = 3, rotated) (*f*_21_)		[−100,100]^*D*^
Composition Function 6 (*n* = 5, rotated) (*f*_22_)		[−100,100]^*D*^
Composition Function 7 (*n* = 5, rotated) (*f*_23_)		[−100,100]^*D*^
Composition Function 8 (*n* = 5, rotated) (*f*_24_)		[−100,100]^*D*^

**Table 2:** Results of HPFA on Sphere with different CR  and *F*.

CR	*F*	Mean	Std	Best	Worst
0.1	0.1	1.804934*E* − 41	4.306427*E* − 41	2.744826*E* − 45	1.913165*E* − 40
0.5	0.1	6.469129*E* − 52	9.396465*E* − 52	3.918891*E* − 54	3.663762*E* − 51
**0.9**	**0.1**	**2.675215*E* − 56**	**7.790529*E* − 56**	**1.508425*E* − 58**	**3.537206*E* − 55**
0.1	0.5	4.133484*E* − 37	9.188120*E* − 37	2.575105*E* − 39	3.202340*E* − 36
0.5	0.5	4.453667*E* − 39	7.464029*E* − 39	6.254230*E* − 42	2.303252*E* − 38
0.9	0.5	3.829022*E* − 38	6.050527*E* − 38	4.375602*E* − 41	2.285076*E* − 37
0.1	0.7	1.657355*E* − 35	3.454283*E* − 35	8.047479*E* − 39	1.298767*E* − 34
0.5	0.7	1.317298*E* − 32	4.014582*E* − 32	2.297207*E* − 36	1.744416*E* − 31
0.9	0.7	3.390563*E* − 31	1.259625*E* − 30	3.333444*E* − 34	5.672141*E* − 30

Values in bold represent the best results.

**Table 3:** Results of HPFA on Zakharov with different CR  and *F*.

CR	F	Mean	Std	Best	Worst
0.1	0.1	8.997799*E* − 05	1.962703*E* − 04	8.098559*E* − 06	8.982455*E* − 04
0.5	0.1	4.735121*E* − 06	6.379299*E* − 06	2.419542*E* − 08	2.150716*E* − 05
**0.9**	**0.1**	**1.525978*E* − 07**	**2.914531*E* − 07**	**1.794753*E* − 09**	**1.294071*E* − 06**
0.1	0.5	1.221220*E* − 04	1.087968*E* − 04	6.458847*E* − 06	3.542003*E* − 04
0.5	0.5	6.305502*E* − 04	6.049323*E* − 04	5.763775*E* − 05	2.276017*E* − 03
0.9	0.5	5.562315*E* − 04	4.954291*E* − 04	4.126934*E* − 05	1.797681*E* − 03
0.1	0.7	8.458769*E* − 04	8.987164*E* − 04	5.839103*E* − 05	3.582266*E* − 03
0.5	0.7	2.174830*E* − 03	2.124124*E* − 03	2.583813*E* − 04	7.311997*E* − 03
0.9	0.7	2.343529*E* − 03	3.139729*E* − 03	2.687660*E* − 04	1.161894*E* − 02

Values in bold represent the best results.

**Table 4:** Results of HPFA on Sumsquares with different CR and *F*.

CR	*F*	Mean	Std	Best	Worst
0.1	0.1	2.730851*E* − 39	5.758742*E* − 39	2.924781*E* − 43	2.605756*E* − 38
0.5	0.1	2.770970*E* − 49	9.552035*E* − 49	1.926855*E* − 52	4.282311*E* − 48
**0.9**	**0.1**	**3.816930*E* − 53**	**1.447266*E* − 52**	**1.874149*E* − 56**	**6.516979*E* − 52**
0.1	0.5	5.870955*E* − 35	9.623223*E* − 35	2.036393*E* − 39	3.519882*E* − 34
0.5	0.5	7.252112*E* − 37	1.091354*E* − 36	6.302606*E* − 40	3.513496*E* − 36
0.9	0.5	1.835751*E* − 35	5.192652*E* − 35	6.229899*E* − 39	2.334590*E* − 34
0.1	0.7	3.693683*E* − 32	1.376861*E* − 31	7.899420*E* − 37	6.173252*E* − 31
0.5	0.7	1.028391*E* − 30	1.761916*E* − 30	8.740890*E* − 34	6.752688*E* − 30
0.9	0.7	6.655358*E* − 30	1.166885*E* − 29	1.417114*E* − 33	4.895754*E* − 29

Values in bold represent the best results.

**Table 5:** Results of HPFA on Quadric with different CR and *F*.

CR	*F*	Mean	Std	Best	Worst
0.1	0.1	2.843327*E* − 06	3.049119*E* − 06	1.156593*E* − 07	1.152089*E* − 05
0.5	0.1	4.829416*E* − 07	7.158196*E* − 07	1.046789*E* − 08	3.167360*E* − 06
**0.9**	**0.1**	**1.872879*E* − 08**	**2.807535*E* − 08**	**4.203582*E* − 10**	**1.287137*E* − 07**
0.1	0.5	8.270814*E* − 06	8.651775*E* − 06	1.341972*E* − 06	3.780385*E* − 05
0.5	0.5	2.117334*E* − 05	3.313434*E* − 05	3.112510*E* − 07	1.115489*E* − 04
0.9	0.5	4.016124*E* − 06	6.668875*E* − 06	1.364465*E* − 07	3.043102*E* − 05
0.1	0.7	1.837195*E* − 05	3.350088*E* − 05	2.420329*E* − 07	1.536438*E* − 04
0.5	0.7	3.035135*E* − 05	4.375764*E* − 05	3.853599*E* − 07	1.721490*E* − 04
0.9	0.7	3.848983*E* − 05	4.027965*E* − 05	1.977449*E* − 06	1.598706*E* − 04

Values in bold represent the best results.

**Table 6:** Result comparison of different optimal algorithms with a dimension of 30.

Function		HPFA	PFA	CPSO	PSO	DE
*f*_1_	Mean	**2.87789*E* − 39**	9.96648*E* − 35	1.03012*E* − 05	1.10504*E* − 05	2.63694*E* − 09
	Std	**4.69565*E* − 39**	2.46385*E* − 34	1.23861*E* − 05	7.87840*E* − 06	7.52717*E* − 10
	Best	**6.43021*E* − 42**	1.33831*E* − 38	2.30640*E* − 06	1.11744*E* − 06	1.49876*E* − 09
	Worst	**2.06766*E* − 38**	1.17598*E* − 33	6.50238*E* − 05	2.92489*E* − 05	4.95148*E* − 09
	Rank	**1**	2	4	5	3
*f*_2_	Mean	2.91086*E* + 01	1.80802*E* + 01	**1.39096*E* + 01**	2.97779*E* + 01	4.07529*E* + 01
	Std	2.07275*E* + 01	5.62894*E* + 00	**2.53192*E* + 01**	1.24243*E* + 01	1.34505*E* + 01
	Best	1.55315*E* + 01	3.11018*E* − 01	**2.62037*E* − 01**	2.26400*E* + 01	2.89945*E* + 01
	Worst	7.62099*E* + 01	2.68566*E* + 01	**7.22219*E* + 01**	9.48405*E* + 01	9.28212*E* + 01
	Rank	3	2	**1**	4	5
*f*_3_	Mean	1.75728*E* − 02	**6.04205*E* − 04**	7.34680*E* + 01	3.49305*E* − 02	1.62253*E* + 02
	Std	1.15332*E* − 02	**4.59306*E* − 04**	3.63038*E* + 01	2.55524*E* − 02	2.26821*E* + 01
	Best	3.93048*E* − 03	**9.02740*E* − 05**	2.52930*E* + 01	8.92958*E* − 03	1.06934*E* + 02
	Worst	4.89242*E* − 02	**1.80294*E* − 03**	1.78108*E* + 02	1.21832*E* − 01	1.98620*E* + 02
	Rank	2	**1**	4	3	5
*f*_4_	Mean	**1.59636*E* − 32**	8.29261*E* − 02	5.94602*E* − 04	6.83859*E* − 01	4.51854*E* − 08
	Std	**6.25035*E* − 34**	1.73018*E* − 01	4.23066*E* − 04	7.90339*E* − 01	1.41403*E* − 08
	Best	**1.57055*E* − 32**	6.08824*E* − 32	1.81251*E* − 04	5.30289*E* − 04	2.13220*E* − 08
	Worst	**1.82870*E* − 32**	6.21900*E* − 01	1.61115*E* − 03	2.64677*E* + 00	8.21182*E* − 08
	Rank	**1**	4	3	5	2
*f*_5_	Mean	**2.15329*E* − 36**	3.79305*E* − 31	6.94271*E* − 04	2.70777*E* − 03	1.41395*E* − 07
	Std	**6.51598*E* − 36**	1.29899*E* − 30	4.38126*E* − 04	1.78871*E* − 03	3.74697*E* − 08
	Best	**1.20023*E* − 38**	3.34744*E* − 35	1.99516*E* − 04	4.14323*E* − 04	6.89551*E* − 08
	Worst	**3.44935*E* − 35**	6.72983*E* − 30	1.99485*E* − 03	7.01576*E* − 03	2.30300*E* − 07
	Rank	**1**	2	4	5	3
*f*_6_	Mean	1.16026*E* + 00	**1.84604*E* − 01**	3.04128*E* + 02	4.64371*E* + 01	1.49977*E* + 02
	Std	5.79662*E* − 01	**1.98144*E* − 01**	7.54896*E* + 01	7.99384*E* + 01	2.08215*E* + 01
	Best	1.65827*E* − 01	**6.50787*E* − 03**	1.79068*E* + 02	1.29523*E* − 02	1.18058*E* + 02
	Worst	2.50218*E* + 00	**8.10651*E* − 01**	4.89106*E* + 02	3.09935*E* + 02	1.91642*E* + 02
	Rank	2	**1**	5	3	4
*f*_7_	Mean	1.14655*E* − 11	3.49179*E* − 10	3.65726*E* − 09	1.26347*E* − 16	**1.47712*E* − 35**
	Std	2.19632*E* − 11	3.45822*E* − 10	6.05869*E* − 09	2.33999*E* − 16	**2.28754*E* − 35**
	Best	2.82583*E* − 18	3.13738*E* − 11	1.06366*E* − 12	6.12039*E* − 19	**3.24107*E* − 37**
	Worst	8.56564*E* − 11	1.35814*E* − 09	2.62745*E* − 08	1.05378*E* − 15	**1.04388*E* − 34**
	Rank	3	4	5	2	**1**
*f*_8_	Mean	**2.81761*E* − 22**	1.00686*E* − 17	2.11152*E* − 02	2.62070*E* − 01	9.04675*E* − 05
	Std	**3.17278*E* − 22**	2.17703*E* − 17	5.45981*E* − 03	2.26377*E* − 01	1.35825*E* − 05
	Best	**1.44549*E* − 23**	3.75280*E* − 19	1.30870*E* − 02	6.06860*E* − 02	6.44440*E* − 05
	Worst	**1.34912*E* − 21**	1.18525*E* − 16	3.44021*E* − 02	1.12460*E* + 00	1.17674*E* − 04
	Rank	**1**	2	4	5	3
*f*_9_	Mean	3.11760*E* + 00	7.85020*E* + 01	**1.67718*E* − 02**	6.84768*E* + 01	1.17019*E* − 01
	Std	1.80524*E* + 00	2.52468*E* + 01	**8.68494*E* − 03**	1.56707*E* + 01	7.47997*E* − 02
	Best	8.90905*E* − 05	2.98487*E* + 01	**8.97848*E* − 03**	3.18446*E* + 01	1.33573*E* − 02
	Worst	6.96471*E* + 00	1.51233*E* + 02	**4.38635*E* − 02**	9.68780*E* + 01	3.11367*E* − 01
	Rank	3	5	**1**	4	2
*f*_10_	Mean	1.30333*E* + 02	3.70867*E* + 03	**4.19844*E* − 03**	6.11047*E* + 03	7.89627*E* + 00
	Std	1.65718*E* + 02	7.73401*E* + 02	**1.59645*E* − 03**	7.25163*E* + 02	3.00488*E* + 01
	Best	3.81827*E* − 04	2.27312*E* + 03	**1.74911*E* − 03**	4.38253*E* + 03	3.82429*E* − 04
	Worst	5.92192*E* + 02	5.17192*E* + 03	**8.19982*E* − 03**	7.32447*E* + 03	1.18439*E* + 02
	Rank	3	4	**1**	5	2
*f*_11_	Mean	**7.87518*E* − 15**	3.66167*E* − 01	1.85383*E* − 02	2.10399*E* + 00	2.98635*E* − 04
	Std	**6.48634*E* − 16**	7.03922*E* − 01	4.72574*E* − 03	4.45303*E* − 01	4.19896*E* − 05
	Best	**4.44089*E* − 15**	7.99361*E* − 15	1.02247*E* − 02	1.15607*E* + 00	2.35379*E* − 04
	Worst	**7.99361*E* − 15**	2.40831*E* + 00	2.85422*E* − 02	3.46435*E* + 00	4.22172*E* − 04
	Rank	**1**	4	3	5	2
*f*_12_	Mean	7.39604*E* − 04	6.89544*E* − 03	2.54495*E* − 02	3.42781*E* − 02	**1.79665*E* − 05**
	Std	2.25674*E* − 03	7.89942*E* − 03	4.01058*E* − 02	1.67595*E* − 02	**1.46270*E* − 05**
	Best	0.00000*E* + 00	0.00000*E* + 00	2.25373*E* − 03	1.14047*E* − 02	**4.39881*E* − 06**
	Worst	7.39604*E* − 03	2.70370*E* − 02	1.59378*E* − 01	9.13585*E* − 02	**6.22646*E* − 05**
	Rank	2	3	4	5	**1**
*f*_13_	Mean	**2.95051*E* + 01**	8.19844*E* + 01	1.48734*E* + 02	8.36924*E* + 01	1.33984*E* + 02
	Std	**1.17090*E* + 01**	3.17715*E* + 01	3.99671*E* + 01	1.83198*E* + 01	8.84993*E* + 00
	Best	**1.29345*E* + 01**	4.07933*E* + 01	8.25970*E* + 01	5.27381*E* + 01	1.11286*E* + 02
	Worst	**5.73477*E* + 01**	1.90036*E* + 02	2.31836*E* + 02	1.38309*E* + 02	1.47944*E* + 02
	Rank	**1**	2	5	3	4
*f*_14_	Mean	**2.34246*E* + 03**	2.66915*E* + 03	4.34728*E* + 03	5.88264*E* + 03	3.56508*E* + 03
	Std	**6.89972*E* + 02**	7.51855*E* + 02	7.16422*E* + 02	8.71985*E* + 02	3.36531*E* + 02
	Best	**1.08765*E* + 03**	1.48280*E* + 03	3.13281*E* + 03	4.61162*E* + 03	2.74852*E* + 03
	Worst	**3.75773*E* + 03**	4.16221*E* + 03	5.66909*E* + 03	7.97547*E* + 03	4.12042*E* + 03
	Rank	**1**	2	4	5	3
*f*_15_	Mean	**7.75676*E* − 15**	1.08189*E* + 00	4.92103*E* + 00	2.63458*E* + 00	1.30715*E* − 01
	Std	**9.01352*E* − 16**	8.67751*E* − 01	6.37440*E* + 00	6.36446*E* − 01	8.99981*E* − 02
	Best	**4.44089*E* − 15**	7.99361*E* − 15	4.19255*E* − 02	1.34065*E* + 00	3.94788*E* − 02
	Worst	**7.99361*E* − 15**	2.57954*E* + 00	1.93777*E* + 01	4.46577*E* + 00	4.41820*E* − 01
	Rank	**1**	3	5	4	2
*f*_16_	Mean	**1.31463*E* − 03**	7.79994*E* − 03	5.19982*E* − 02	3.83314*E* − 02	1.03911*E* − 02
	Std	**3.46976*E* − 03**	8.35854*E* − 03	4.49126*E* − 02	2.35712*E* − 02	6.99527*E* − 03
	Best	**0.00000*E* + 00**	0.00000*E* + 00	5.47885*E* − 03	6.25370*E* − 03	1.97137*E* − 03
	Worst	**1.23210*E* − 02**	2.70517*E* − 02	1.76100*E* − 01	1.15044*E* − 01	2.79564*E* − 02
	Rank	**1**	2	5	4	3
*f*_17_	Mean	9.94354*E* + 02	9.97257*E* + 02	**9.87240*E* + 02**	9.98219*E* + 02	1.02307*E* + 03
	Std	6.45565*E* + 01	7.93940*E* + 01	**8.18461*E* + 01**	8.71585*E* + 01	9.06629*E* + 01
	Best	9.00000*E* + 02	9.00000*E* + 02	**8.02234*E* + 02**	8.02117*E* + 02	9.00224*E* + 02
	Worst	1.14354*E* + 03	1.14354*E* + 03	**1.14355*E* + 03**	1.14355*E* + 03	1.15475*E* + 03
	Rank	2	3	**1**	4	5
*f*_18_	Mean	2.16969*E* + 03	4.27549*E* + 03	**9.23935*E* + 02**	5.68722*E* + 03	1.75920*E* + 03
	Std	5.31124*E* + 02	6.06241*E* + 02	**6.72922*E* + 01**	8.46528*E* + 02	2.19741*E* + 02
	Best	1.52934*E* + 03	3.04609*E* + 03	**8.00563*E* + 02**	3.78428*E* + 03	1.38322*E* + 03
	Worst	3.71231*E* + 03	5.73560*E* + 03	**1.03826*E* + 03**	7.46332*E* + 03	2.24557*E* + 03
	Rank	3	4	**1**	5	2
*f*_19_	Mean	6.49831*E* + 03	**5.86482*E* + 03**	7.17359*E* + 03	6.28350*E* + 03	7.96975*E* + 03
	Std	8.70510*E* + 02	**5.57598*E* + 02**	9.53474*E* + 02	7.93415*E* + 02	4.27488*E* + 02
	Best	4.52076*E* + 03	**4.60223*E* + 03**	5.58118*E* + 03	4.29488*E* + 03	7.13518*E* + 03
	Worst	7.85971*E* + 03	**6.85501*E* + 03**	8.76360*E* + 03	7.70667*E* + 03	8.76090*E* + 03
	Rank	3	**1**	4	2	5
*f*_20_	Mean	**1.24508*E* + 03**	1.26066*E* + 03	1.31885*E* + 03	1.29748*E* + 03	1.28362*E* + 03
	Std	**8.94977*E* + 00**	1.29159*E* + 01	1.40407*E* + 01	1.35512*E* + 01	4.72764*E* + 00
	Best	**1.22474*E* + 03**	1.23950*E* + 03	1.28952*E* + 03	1.27569*E* + 03	1.27268*E* + 03
	Worst	**1.26015*E* + 03**	1.29628*E* + 03	1.35574*E* + 03	1.31989*E* + 03	1.29134*E* + 03
	Rank	**1**	2	5	4	3
*f*_21_	Mean	**1.35640*E* + 03**	1.37460*E* + 03	1.43436*E* + 03	1.42631*E* + 03	1.39210*E* + 03
	Std	**7.04293*E* + 00**	1.08266*E* + 01	1.53275*E* + 01	1.47903*E* + 01	4.02294*E* + 00
	Best	**1.33796*E* + 03**	1.35580*E* + 03	1.40858*E* + 03	1.40163*E* + 03	1.38252*E* + 03
	Worst	**1.36691*E* + 03**	1.39966*E* + 03	1.47166*E* + 03	1.45863*E* + 03	1.39765*E* + 03
	Rank	**1**	2	5	4	3
*f*_22_	Mean	1.47852*E* + 03	1.49776*E* + 03	1.55974*E* + 03	1.50353*E* + 03	**1.40407*E* + 03**
	Std	6.98737*E* + 01	7.59405*E* + 01	8.14332*E* + 01	8.63327*E* + 01	**9.84591*E* − 01**
	Best	1.40006*E* + 03	1.40007*E* + 03	1.40033*E* + 03	1.40002*E* + 03	**1.40219*E* + 03**
	Worst	1.54609*E* + 03	1.57282*E* + 03	1.61942*E* + 03	1.58723*E* + 03	**1.40652*E* + 03**
	Rank	2	3	5	4	**1**
*f*_23_	Mean	**2.02871*E* + 03**	2.17259*E* + 03	2.65148*E* + 03	2.39185*E* + 03	2.41610*E* + 03
	Std	**7.62593*E* + 01**	1.12942*E* + 02	1.02039*E* + 02	1.10303*E* + 02	5.57424*E* + 01
	Best	**1.88030*E* + 03**	1.97638*E* + 03	2.34650*E* + 03	2.16373*E* + 03	2.20448*E* + 03
	Worst	**2.16629*E* + 03**	2.41923*E* + 03	2.79679*E* + 03	2.60090*E* + 03	2.49332*E* + 03
	Rank	**1**	2	5	3	4
*f*_24_	Mean	**1.70000*E* + 03**	1.78188*E* + 03	3.65173*E* + 03	3.10618*E* + 03	1.70163*E* + 03
	Std	**3.32458*E* − 13**	3.11801*E* + 02	1.46943*E* + 03	1.25346*E* + 03	3.11041*E* + 00
	Best	**1.70000*E* + 03**	1.70000*E* + 03	1.70448*E* + 03	1.50128*E* + 03	1.70046*E* + 03
	Worst	**1.70000*E* + 03**	2.97117*E* + 03	7.92326*E* + 03	4.85875*E* + 03	1.71777*E* + 03
	Rank	**1**	3	5	4	2

**Table 7:** Results of the Iman–Davenport test.

Dimension	Iman–Davenport	Critical value *α* = 0.05	Significant differences?
30	11.863	2.45∼2.52	Yes

**Table 8:** Comparison (Holm's test) of HPFA with the remaining algorithms.

Algorithm	*z*	*p* value	*α*/*i*	Significant differences?
PSO	5.1121	3.1863*E* − 7	0.0125	Yes
CPSO	4.3817	1.1771*E* − 5	0.0167	Yes
DE	2.6473	0.00811	0.025	Yes
PFA	2.0083	0.0446	0.05	Yes

**Table 9:** Mean total within-cluster variances of HPFA, PFA, CPSO, and PSO algorithms.

Datasets		HPFA	PFA	PSO	CPSO
Glass	Mean	**235.67**	238.84	510.58	2438.13
	Std	**15.06**	13.35	58.75	86.37
	Rank	**1**	2	3	4
Wine	Mean	**16293.27**	16293.44	18345.01	17754.60
	Std	**0.86**	0.83	1017.12	1011.00
	Rank	**1**	2	4	3
Iris	Mean	**97.64**	97.69	159.33	161.88
	Std	**4.36**	5.66	18.25	17.21
	Rank	**1**	2	3	4
LD	Mean	**9851.84**	9851.87	13760.02	15357.00
	Std	**0.19**	0.17	1457.21	1667.36
	Rank	**1**	2	3	4

**Table 10:** Comparison of the best solution obtained from the previous algorithms for constrained problem 1.

DV	IGA	WCA	HPFA	Optimal solution
*x*_1_	2.330499	2.334238	2.330704	2.330499
*x*_2_	1.951372	1.950249	1.953073	1.951372
*x*_3_	−0.477541	−0.474707	−0.476937	−0.477541
*x*_4_	4.365726	4.366854	4.361683	4.365726
*x*_5_	−0.624487	−0.619911	−0.627155	−0.624487
*x*_6_	1.038131	1.030*E* + 00	1.037494	1.038131
*x*_7_	1.594227	1.595*E* + 00	1.593695	1.594227
*g*1(*x*)	4.46000*E* − 05	1.00000*E* − 13	−1.00000*E* − 04	4.46000*E* − 05
*g*2(*x*)	−252.561723	252.569346	−252.5623	252.561723
*g*3(*x*)	−144.878190	144.897817	−144.8705	−144.87819
*g*4(*x*)	7.63000*E* − 06	2.2*E* − 12	−0.0011	7.63000*E* − 06
*f* (*x*)	680.630060	680.631178	680.631176	680.630057

**Table 11:** Comparison of statistical results obtained from various algorithms for constrained problem 1.

Methods	Mean	SD	Best	Worst	FEs
HPFA	680.6338	1.835611*E* − 03	680.6312	680.6376	100000
WCA	680.6443	1.140000*E* − 02	680.6311	680.6738	110050
PSO	680.9710	5.100000*E* − 01	680.6345	684.5289	140100
CPSO-GD	680.7810	1.484000*E* − 01	680.6780	681.3710	NA
CDE	681.5030	NA	680.7710	685.1440	248000

**Table 12:** Comparison of the best solution obtained from the previous algorithms for constrained problem 2.

DV	GA1	WCA	HPFA	Optimal solution
*x*_1_	78.049500	78.000000	78.000000	78.000000
*x*_2_	33.007000	33.000000	33.000000	33.000000
*x*_3_	27.081000	29.995256	29.995256	29.995260
*x*_4_	45.000000	45.000000	45.000000	45.000000
*x*_5_	44.940000	36.775812	36.775813	36.775810
*g*1(*x*)	1.284*E* + 00	−1.960*E* − 12	0	−9.7100*E* − 04
*g*2(*x*)	−93.283813	−91.999999	−92.000000	−92.000000
*g*3(*x*)	−9.592143	−11.159499	−11.159500	−11.100000
*g*4(*x*)	−10.407856	−8.840500	−8.840500	−8.870000
*g*5(*x*)	−4.998088	−5.000000	−5.000000	−5.000000
*g*6(*x*)	1.910000000*E* − 03	0	0	9.27*E* − 09
*f*(*x*)	−31020.859000	−30665.538600	−30665.538600	−30665.539000

**Table 13:** Comparison of statistical results obtained from various algorithms for constrained problem 2.

Methods	Mean	SD	Best	Worst	FEs
HPFA	−30665.5380	6.365971*E* − 04	−30665.5386	−30665.5354	15000
WCA	−30665.5270	2.180000*E* − 02	−30665.5386	−30665.4570	18850
PSO	−30570.9286	8.100000*E* + 01	−30663.8563	−30252.3258	70100
HPSO	−30665.5390	1.700000*E* − 06	−30665.5390	−30665.5390	81000
PSO-DE	−30665.5387	8.300000*E* − 10	−30665.5387	−30665.5387	70100
DE	−30665.5360	5.067000*E* − 03	−30665.5390	−30665.5090	240000

**Table 14:** Comparison of the best solution obtained from the previous algorithms for constrained problem 3.

DV	CULDE	WCA	HPFA	Optimal solution
*x*_1_	0.304887	0.316011	0.316209	0.316227
*x*_2_	0.329917	0.316409	0.315936	0.316227
*x*_3_	0.319260	0.315392	0.315973	0.316227
*x*_4_	0.328069	0.315872	0.316330	0.316227
*x*_5_	0.326023	0.316570	0.316688	0.316227
*x*_6_	0.302707	0.316209	0.315952	0.316227
*x*_7_	0.305104	0.316137	0.315959	0.316227
*x*_8_	0.315312	0.316723	0.315921	0.316227
*x*_9_	0.322047	0.316924	0.316752	0.316227
*x*_10_	0.309009	0.316022	0.316555	0.316227
*h*(*x*)	9.910000000*E* − 04	0	−6.86*E* − 07	0
*f*(*x*)	−0.995413	−0.999981	−0.999989	−1.000000

**Table 15:** Comparison of statistical results obtained from various algorithms for constrained problem 3.

Methods	Mean	SD	Best	Worst	FEs
HPFA	−0.999956	1.827886*E* − 05	−0.999989	−0.999918	100000
WCA	−0.999806	1.910000*E* − 04	−0.999981	−0.999171	103900
PSO	−1.004879	1.000000E + 00	−1.004986	−1.004269	140100
PSO-DE	−1.005010	3.800000*E* − 12	−1.005010	−1.005010	140100
DE	−1.025200	NA	−1.025200	−1.025200	8000000

**Table 16:** Comparison of the best solution obtained from the previous algorithms for constrained problem 4.

DV	WCA	HPFA	Optimal solution
*x*_1_	5.000000	5.000000	5.000000
*x*_2_	5.000000	5.000000	5.000000
*x*_3_	5.000000	5.000000	5.000000
*g*1(*x*)	47.937496	47.937501	47.937500
*g*2(*x*)	26.937497	26.937501	26.937500
*g*3(*x*)	11.937498	11.937501	11.937500
*g*4(*x*)	2.937499	2.937500	2.937500
*g*5(*x*)	−0.062500	−0.062500	−0.062500
*g*6(*x*)	2.937501	2.937500	2.937500
*g*7(*x*)	11.937502	11.937499	11.937500
*g*8(*x*)	26.937503	26.937499	26.937500
*g*9(*x*)	47.937504	47.937499	47.937500
*f*(*x*)	−9.999990*E* − 01	−1.0000000	−1.000000

**Table 17:** Comparison of statistical results obtained from various algorithms for constrained problem 4.

Methods	Mean	SD	Best	Worst	FEs
HPFA	−1.000000	7.358561*E* − 15	−1.000000	−1.000000	5000
WCA	−0.999999	2.510000*E* − 07	−0.999999	−0.999998	6100
HPSO	−1.000000	1.600000*E* − 15	−1.000000	−1.000000	81000
PESO	−0.998875	NA	−1.000000	−0.994000	350000
TLBO	−1.000000	0.000000*E* + 00	−1.000000	−1.000000	50000

**Table 18:** Comparison of the best solution obtained from the previous algorithms for the three-bar truss design problem.

DV	PSO-DE	WCA	HPFA
*x*_1_	0.788675	0.788651	0.788674
*x*_2_	0.408248	0.408316	0.408251
*g*1(*x*)	−5.290000*E* − 11	0	3.981300*E* − 11
*g*2(*x*)	−1.463747	−1.464024	−1.464098
*g*3(*x*)	−0.536252	−0.535975	−0.535902
*f*(*x*)	263.895843	263.895843	263.895843

**Table 19:** Comparison of statistical results obtained from various algorithms for the three-bar truss design problem.

Methods	Mean	SD	Best	Worst	FEs
HPFA	263.895942	2.300924*E* − 04	263.895843	263.896833	10000
WCA	263.895903	8.710000*E* − 05	263.895843	263.896201	5250
PSO-DE	263.895843	4.500000*E* − 10	263.895843	263.895843	17600

**Table 20:** Comparison of the best solution obtained from the previous algorithms for the speed reducer problem.

DV	WCA	HPFA	PSO-DE	HEAA
*x*_1_	3.500000	3.500000	3.500000	3.500022
*x*_2_	0.700000	0.700000	0.700000	0.700000
*x*_3_	17.000000	17.000001	17.000000	17.000012
*x*_4_	7.300000	7.300002	7.300000	7.300427
*x*_5_	7.715319	7.715321	7.800000	7.715377
*x*_6_	3.350214	3.350215	3.350214	3.350230
*x*_7_	5.286654	5.286655	5.286683	5.286663
*f*(*x*)	2994.471066	2994.471705	2996.348167	2994.499107

**Table 21:** Comparison of statistical results obtained from various algorithms for the speed reducer problem.

Methods	Mean	SD	Best	Worst	FEs
HPFA	2994.473059	1.005309*E* − 03	2994.471705	2994.475855	11000
WCA	2994.474392	7.400000*E* − 03	2994.471066	2994.505578	15150
PSO-DE	2996.348174	6.400000*E* − 06	2996.348167	2996.348204	54350
HEAA	2994.613368	7.000000*E* − 02	2994.499107	2994.752311	40000

**Table 22:** Comparison of the best solution obtained from the previous algorithms for the pressure vessel problem.

DV	WCA	HPFA	CPSO	GA3
*x*_1_	0.778100	0.778547	0.812500	0.812500
*x*_2_	0.384600	0.384828	0.437500	0.437500
*x*_3_	40.319600	40.338244	42.091300	42.097400
*x*_4_	-200.000000	199.759935	176.746500	176.654000
*g*1(*x*)	−2.950000*E* − 11	−1.89*E* − 05	−1.370000*E* − 06	−2.010000*E* − 03
*g*2(*x*)	−7.150000*E* − 11	−1.15*E* − 06	−3.590000*E* − 04	−3.580000*E* − 02
*g*3(*x*)	−1.350000*E* − 06	−97.382360	−118.768700	−24.759300
*g*4(*x*)	−40.000000	−40.240060	−63.253500	−63.346000
*f*(*x*)	5885.332700	5886.495946	6061.077700	6059.946300

**Table 23:** Comparison of statistical results obtained from various algorithms for the pressure vessel problem.

Methods	Mean	SD	Best	Worst	FEs
HPFA	6321.480545	3.565060*E* + 02	5886.495946	7106.967827	25000
WCA	6198.617200	2.130490*E* + 02	5885.332700	6590.212900	27500
CPSO	6147.133200	8.645000*E* + 01	6061.077700	6363.804100	240000
PSO	8756.680300	1.492567*E* + 03	6693.721200	14076.324000	8000

**Table 24:** Comparison of the best solution obtained from the previous algorithms for the tension compression spring problem.

DV	WCA	HPFA	CPSO	GA3
*x*_1_	0.051680	0.051536	0.051728	0.051989
*x*_2_	0.356522	0.353035	0.357644	0.363965
*x*_3_	11.300410	11.508944	11.244543	10.890522
*g*1(*x*)	−1.650000*E* − 13	−1.23442*E* − 05	−8.250000*E* − 04	−1.260000*E* − 03
*g*2(*x*)	−7.900000*E* − 14	−3.65699*E* − 05	−2.520000*E* − 05	−2.540000*E* − 05
*g*3(*x*)	−4.053399	−4.046190	−4.051306	−4.061337
*g*4(*x*)	−0.727864	−0.730286	−0.727085	−0.722697
*f*(*x*)	0.012665	0.012667	0.012674	0.012681

**Table 25:** Comparison of statistical results obtained from various algorithms for the tension compression spring problem.

Methods	Mean	SD	Best	Worst	FEs
HPFA	0.012727	7.707958*E* − 05	0.012667	0.013018	22000
WCA	0.012746	8.060000*E* − 05	0.012665	0.012952	11750
CPSO	0.012730	5.200000*E* − 04	0.012674	0.012924	240000
GA3	0.012742	5.900000*E* − 05	0.012681	0.012973	80000

**Table 26:** Comparison of the best solution obtained from the previous algorithms for the welded beam problem.

DV	WCA	HPFA	CPSO	GA3
*x*_1_	0.205728	0.205730	0.202369	0.205986
*x*_2_	3.470522	3.470490	3.544214	3.471328
*x*_3_	9.036620	9.036623	9.048210	9.020224
*x*_4_	0.205729	0.205730	0.205723	0.206480
*g*1(*x*)	−0.034128	−0.004198	−13.655547	−0.103049
*g*2(*x*)	−0.000035	−0.004067	−78.814077	−0.231747
*g*3(*x*)	−0.000001	0.000000	−0.003500	−0.000050
*g*4(*x*)	−3.432980	−3.432983	−3.424572	−3.430044
*g*5(*x*)	−0.080728	−0.080730	−0.077369	−0.080986
*g*6(*x*)	−0.235540	−0.235540	−0.235595	−0.235514
*g*7(*x*)	−0.013503	−0.003946	−4.472858	−58.646888
*f*(*x*)	1.724856	1.724853	1.728024	1.728226

**Table 27:** Comparison of statistical results obtained from various algorithms for the welded beam problem.

Methods	Mean	SD	Best	Worst	FEs
HPFA	1.724889	1.027784*E* − E-04	1.724853	1.725354	22000
WCA	1.726427	4.290000*E* − 03	1.724856	1.744697	46450
CPSO	1.748831	1.290000*E* − 02	1.728024	1.782143	240000
GA3	1.792654	7.470000*E* − 02	1.728226	1.993408	80000

## Data Availability

Data for clustering in this study have been taken from the UCI machine learning repository (http://archive.ics.uci.edu/ml/index.php). Data are provided freely for academic research purposes only.

## Appendix

### A. Constrained Problem 1

(A.1) $$\begin{array}{c} f\left(x\right)={\left(x_{1}-10\right)}^{2}+5{\left(x_{2}-12\right)}^{2}+x_{3}^{4}+3{\left(x_{4}-11\right)}^{2}+10x_{5}^{6}+7x_{6}^{2}+x_{7}^{4}-4x_{6}x_{7}-10x_{6}-8x_{7}, \end{array}$$

subject to

(A.2) $$\begin{array}{c} g_{1}\left(x\right)=127-2x_{1}^{2}-3x_{2}^{4}-x_{3}-4x_{4}^{2}-5x_{5}\geq 0,\\ g_{2}\left(x\right)=282-7x_{1}-3x_{2}-10x_{3}^{2}-x_{4}+x_{5}\geq 0,\\ g_{3}\left(x\right)=196-23x_{1}-x_{2}^{2}-6x_{6}^{2}+8x_{7}\geq 0,\\ g_{4}\left(x\right)=-4x_{1}^{2}-x_{2}^{2}+3x_{1}x_{2}-2x_{3}^{2}-5x_{6}+11x_{7}\geq 0,\\ -10\leq x_{i}\leq 10,\;i=1,2,3,4,5,6,7. \end{array}$$

### B. Constrained Problem 2

(B.1) $$\begin{array}{c} f\left(x\right)=5.3578547x_{3}^{3}+0.8356891x_{1}x_{5}+37.293239x_{1}+40729.141, \end{array}$$

subject to

(B.2) $$\begin{array}{c} g_{1}\left(x\right)=85.334407+0.0056858x_{2}x_{5}+0.0006262x_{1}x_{4}-0.0022053x_{3}x_{5}-92\leq 0,\\ g_{2}\left(x\right)=-85.334407-0.0056858x_{2}x_{5}-0.0006262x_{1}x_{4}-0.0022053x_{3}x_{5}\leq 0,\\ g_{3}\left(x\right)=80.51249+0.0071317x_{2}x_{5}+0.0029955x_{1}x_{2}+0.00218x_{3}^{2}-110\leq 0,\\ g_{4}\left(x\right)=-80.51249-0.0071317x_{2}x_{5}-0.0029955x_{1}x_{2}-0.0021813x_{3}^{2}+90\leq 0,\\ g_{5}\left(x\right)=9.300961+0.0047026x_{3}x_{5}+0.0012547x_{1}x_{3}+0.0019085x_{3}x_{4}-25\leq 0,\\ g_{6}\left(x\right)=-9.300961-0.0047026x_{3}x_{5}-0.0012547x_{1}x_{3}-0.0019085x_{3}x_{4}+20\leq 0,\\ 78\leq x_{1}\leq 102,\;33\leq x_{2}\leq 45,\;27\leq x_{i}\leq 45,\;i=3,4,5. \end{array}$$

### C. Constrained Problem 3

(C.1) $$\begin{array}{c} f\left(x\right)=-{\left(\sqrt{n}\right)}^{n}\prod_{i=1}^{n}x_{i}, \end{array}$$

subject to

(C.2) $$\begin{array}{c} h\left(x\right)=\sum_{i=1}^{n}x_{i}^{2}=1,\;0\leq x_{i}\leq 1,i=1,\ldots ,n. \end{array}$$

### D. Constrained Problem 4

(D.1) $$\begin{array}{c} f\left(x\right)=-\frac{100-{\left(x_{1}-5\right)}^{2}-{\left(x_{2}-5\right)}^{2}-{\left(x_{3}-5\right)}^{2}}{100}, \end{array}$$

subject to

(D.2) $$\begin{array}{c} g\left(x\right)={\left(x_{1}-p\right)}^{2}+{\left(x_{2}-q\right)}^{2}+{\left(x_{3}-r\right)}^{2}-0.0625\leq 0,\;0\leq x_{i}\leq 10\;i=1,2,3\;p,q,r=1,2,3,\ldots ,9. \end{array}$$

### E. Three-Bar Truss Design Problem

(E.1) $$\begin{array}{c} f\left(x\right)=\left(2\sqrt{2}x_{1}+x_{2}\right)\times l, \end{array}$$

subject to

(E.2) $$\begin{array}{c} g_{1}\left(x\right)=\frac{\sqrt{2}x_{1}+x_{2}}{\sqrt{2}x_{1}^{2}+2x_{1}x_{2}}P-\sigma \leq 0,\\ g_{2}\left(x\right)=\frac{x_{2}}{\sqrt{2}x_{1}^{2}+2x_{1}x_{2}}P-\sigma \leq 0,\\ g_{3}\left(x\right)=\frac{1}{\sqrt{2}x_{2}+x_{1}}P-\sigma \leq 0,\\ 0\leq x_{i}\leq 1,\;i=1,2,\\ l=100\text{ cm},\\ P=2\;\text{kN}/{\text{cm}}^{2},\\ \sigma =2\;\text{kN}/{\text{cm}}^{2} \end{array}$$

### F. Speed Reducer Design Problem

(F.1) $$\begin{array}{c} f\left(x\right)=0.785x_{1}x_{2}^{2}\left(3.3333x_{3}^{2}+14.9334x_{3}-43.0934\right)-1.508x_{1}\left(x_{6}^{2}+x_{7}^{2}\right)+7.4777\left(x_{6}^{3}+x_{7}^{3}\right)+0.7854\left(x_{4}x_{6}^{2}+x_{5}x_{7}^{2}\right), \end{array}$$

subject to

(F.2) $$\begin{array}{c} g_{1}\left(x\right)=\frac{27}{x_{1}x_{2}^{2}x_{3}}-1\leq 0,\\ g_{2}\left(x\right)=\frac{397.5}{x_{1}x_{2}^{2}x_{3}^{2}}-1\leq 0,\\ g_{3}\left(x\right)=\frac{1.93x_{4}^{3}}{x_{2}x_{6}^{4}x_{3}}-1\leq 0,\\ g_{4}\left(x\right)=\frac{1.93x_{5}^{3}}{x_{2}x_{7}^{4}x_{3}}-1\leq 0,\\ g_{5}\left(x\right)=\frac{{\left[{\left(745x_{4}/x_{2}x_{3}\right)}^{2}+16.9\times 10^{6}\right]}^{1/2}}{110x_{6}^{3}}-1\leq 0,\\ g_{6}\left(x\right)=\frac{{\left[{\left(745x_{4}/x_{2}x_{3}\right)}^{2}+157.5\times 10^{6}\right]}^{1/2}}{85x_{7}^{3}}-1\leq 0,\\ g_{7}\left(x\right)=\frac{x_{2}x_{3}}{40}-1\leq 0,\\ g_{8}\left(x\right)=\frac{5x_{2}}{x_{1}}-1\leq 0,\\ g_{9}\left(x\right)=\frac{x_{1}}{12x_{2}}-1\leq 0,\\ g_{10}\left(x\right)=\frac{1.5x_{6}+1.9}{x_{4}}-1\leq 0,\\ g_{11}\left(x\right)=\frac{1.1x_{7}+1.9}{x_{5}}-1\leq 0, \end{array}$$

where

(F.3) $$\begin{array}{c} 2.6\leq x_{1}\leq 3.6,0.7\leq x_{2}\leq 0.8,17\leq x_{3}\leq 28,7.3\leq x_{4}\leq 8.3,7.3\leq x_{5}\leq 8.3,2.9\leq x_{6}\leq 3.9,5.0\leq x_{7}\leq 5.5. \end{array}$$

### G. Pressure Vessel Design Problem

(G.1) $$\begin{array}{c} f\left(x\right)=0.6224x_{1}x_{3}x_{4}+1.7781x_{2}x_{3}^{2}+3.1661x_{1}^{2}x_{4}+19.84x_{1}^{2}x_{3}, \end{array}$$

subject to

(G.2) $$\begin{array}{c} g_{1}\left(x\right)=-x_{1}+0.0193x\leq 0,\\ g_{2}\left(x\right)=-x_{2}+0.00954x_{3}\leq 0,\\ g_{3}\left(x\right)=-\pi x_{3}^{2}x_{4}-\frac{4}{3\pi x_{3}^{3}}+1296,000\leq 0,\\ g_{4}\left(x\right)=x_{4}-240\leq 0,\\ 0\leq x_{i}\leq 100,\;i=1,2,\\ 10\leq x_{i}\leq 200,\;i=3,4. \end{array}$$

### H. Tension/Compression Spring Design Problem

(H.1) $$\begin{array}{c} f\left(x\right)=\left(x_{3}+2\right)x_{2}x_{1}^{2}, \end{array}$$

subject to

(H.2) $$\begin{array}{c} g_{1}\left(x\right)=1-\frac{x_{2}^{3}x_{3}}{71,785x_{1}^{4}x_{1}^{4}}\leq 0,\\ g_{2}\left(x\right)=\frac{4x_{2}^{2}-x_{1}x_{2}}{12,566\left(x_{2}x_{1}^{3}-x_{1}^{4}\right)}\;+\frac{1}{5108x_{1}^{2}}-1\leq 0,\\ g_{3}\left(x\right)=1-\frac{140.45x_{1}}{x_{2}^{2}x_{3}}\leq 0,\\ g_{4}\left(x\right)=\frac{x_{2}+x_{1}}{1.5}-1\leq 0,\\ 0.05\leq x_{1}\leq 2.00,\\ 0.25\leq x_{2}\leq 1.30,\\ 2.00\leq x_{3}\leq 15.00. \end{array}$$

### I. Welded Beam Design Problem

(I.1) $$\begin{array}{c} f\left(x\right)=1.10471x_{1}^{2}x_{2}+0.04811x_{3}x_{4}\left(14+x_{2}\right), \end{array}$$

subject to

(I.2) $$\begin{array}{c} g_{1}\left(x\right)=\tau \left(x\right)-\tau_{\max}\leq 0,\\ g_{2}\left(x\right)=\sigma \left(x\right)-\sigma_{\max}\leq 0,\\ g_{3}\left(x\right)=x_{1}-x_{4}\leq 0,\\ g_{4}\left(x\right)=0.10471x_{1}^{2}+0.04811x_{3}x_{4}\left(14+x_{2}\right)-5\leq 0,\\ g_{5}\left(x\right)=0.125-x_{1}\leq 0,\\ g_{6}\left(x\right)=\delta \left(x\right)-\delta_{\max}\leq 0,\\ g_{7}\left(x\right)=P-P_{c}\left(x\right)\leq 0,\\ 0.1\leq x_{i}\leq 2,\;i=1,4,\\ 0.1\leq x_{i}\leq 10,\;i=2,3. \end{array}$$

where,

(I.3) $$\begin{array}{c} \tau \left(x\right)=\sqrt{{\left(\tau^{\prime }\right)}^{2}+2\tau^{\prime }\tau^{\prime \prime }\frac{x_{2}}{2R}+{\left(\tau^{\prime \prime }\right)}^{2}},\;\tau^{\prime }=\frac{P}{\sqrt{2}x_{1}x_{2}},\tau^{\prime \prime }=\frac{\text{MR}}{J},\\ M=P\left(L+\frac{x_{2}}{2}\right),\;\\ R=\sqrt{\frac{x_{2}^{2}}{4}+{\left(\frac{x_{1}+x_{3}}{2}\right)}^{2}},\\ J=2\left\{\sqrt{2}x_{1}x_{2}\left[\frac{x_{2}^{2}}{12}+{\left(\frac{x_{1}+x_{3}}{2}\right)}^{2}\right]\right\},\\ \sigma \left(x\right)=\frac{6PL}{x_{4}x_{3}^{2}},\\ \delta \left(x\right)=\frac{4PL^{3}}{Ex_{3}^{3}x_{4}},\;\\ P_{c}\left(x\right)=\frac{4.013E\sqrt{x_{3}^{2}x_{4}^{6}/36}}{L^{2}}\left(1-\frac{x_{3}}{2L}\sqrt{\frac{E}{4G}}\right),\\ P=6000\text{ lb},\\ L=14\text{ in},\\ \;E=30\times 10^{6}\text{ psi},\\ G=12\times 10^{6}\text{ psi,}\\ \tau_{\max}=13,600\text{ psi},\\ \sigma_{\max}=30,000\text{ psi},\\ \;\delta_{\max}=0.25\;\mathrm{in}. \end{array}$$
